# 1456. Outbreak of *Elizabethkingia anophelis* on a Pulmonary Medicine Unit

**DOI:** 10.1093/ofid/ofad500.1293

**Published:** 2023-11-27

**Authors:** Rachel Weber, Grace Barajas, Sandra Reiner, Michael Malczynski, Chao Qi, Teresa Zembower

**Affiliations:** Northwestern Memorial Hospital, Chicago, Illinois; Northwestern Memorial Hospital, Chicago, Illinois; Northwestern Memorial Hospital, Chicago, Illinois; Northwestern Memorial Hospital, Northwestern University Feinberg School of Medicine, Chicago, Illinois; Northwestern University Feinberg School of Medicine, Northwestern Memorial Hospital, Chicago, IL; Northwestern University, Chicago, Illinois

## Abstract

**Background:**

*Elizabethkingia* species are multidrug-resistant emerging pathogens that have caused outbreaks in healthcare settings and are associated with high mortality rates (20-40%) among infected patients. We experienced an increased number of *E. anophelis* isolates on our high-risk pulmonary medicine unit (PMU) in October 2022. An outbreak investigation was performed.

**Methods:**

The outbreak investigation consisted of review of clinical and microbiological data, evaluation for potential laboratory contamination, water and medical equipment sampling, staff interviews, and pulsed-field gel electrophoresis (PFGE) to characterize clinical and environmental isolates.

**Results:**

Between October and December 2022, *E. anophelis* was identified from respiratory cultures of five colonized patients on our high-risk PMU. None developed clinical infection. By PFGE, three isolates were identical, one was possibly related and one was distinct and not considered part of the outbreak. Laboratory contamination was ruled out when rounds identified no sources and repeat tracheal aspirate cultures processed manually grew *E. anophelis*. Between 10/23/22-10/31/22, all four patients had tracheostomy collars and received respiratory care on overlapping days on PMU. 144 environmental samples were obtained on PMU and three other units where patients had received care: 75 water fixture, 45 water, 15 respiratory equipment, and 9 other medical equipment. 3/144 (2%) environmental samples were positive, all from water fixture samples from PMU. These were distinct from each other and patient isolates. Rounds and interviews identified supplies stored in splash zones, excessive splashing of sinks, and potential misuse of tap water (i.e. rinsing nebulizers). Additionally, PMU had medical equipment needs which resulted in increased sharing of equipment between patients.

**Conclusion:**

Although a definitive source for this outbreak was not found, the investigation concluded that transmissions likely occurred due to tap water misuse coupled with suboptimal infection prevention practices during respiratory procedures. Rapid identification and investigation of this outbreak, including swift awareness, education, and participation of frontline staff, likely contributed to the timely end of this outbreak.

**Disclosures:**

**All Authors**: No reported disclosures

